# ALL HANDS ON DECK: CONCEPTUALIZING AND OPERATIONALIZING COLLABORATION WITHIN CARE NETWORKS

**DOI:** 10.1093/geroni/igac059.1146

**Published:** 2022-12-20

**Authors:** Athena Koumoutzis, Katrina Ellis, Jordan Lewis, Zhiyong Lin, Yuanjin Zhou, William Chopik, Richard Gonzalez

**Affiliations:** Miami University, Oxford, Ohio, United States; University of Michigan, Ann Arbor, Michigan, United States; University of Minnesota Medical School, Duluth, Minnesota, United States; University of Texas at San Antonio, San Antonio, Texas, United States; University of Texas at Austin, Austin, Texas, United States; Michigan State University, East Lansing, Michigan, United States; University of Michigan, Ann Arbor, Michigan, United States

## Abstract

Care recipients often report multiple caregivers that provide assistance. Yet, consideration of conceptual and methodological issues of caregiving networks has yet to be fully explored. This paper proposes a care collaboration index for each care network that predicts variation in collaboration among multiple networks. The association between network size, race/ethnicity, and dementia status with collaboration was also examined. Data came from the 2015 waves of NHATS and NSOC. Operationalization of collaboration was derived from 1,298 caregivers within 552 care networks. Care recipients were older adults (Mage = 83.69, SD = 7.73), most were women (71.6%), 47.9% had possible/probable dementia, and 38.9% identified as persons of color. The collaboration index considered shared care tasks and scope of assistance while controlling for the size of the immediate and broader care networks. This measure also considered task overlap among care networks as a collaboration process. A series of regression models were run to analyze whether care collaboration differed for older adults by key predictors and if the association between care collaboration and predictors varied across care tasks. Care networks with more caregivers garnered greater collaboration overall, both in general and across most types of tasks. Greater collaboration was observed among Black, Hispanic, and Other (non-White) care recipients and those with possible/probable dementia. This index provides a way to examine care network behaviors and highlights the importance of collaboration-informed approaches. Implications regarding the relationship between care collaboration and outcomes for caregivers and recipients will be discussed.

